# Effect of Reflectance Confocal Microscopy for Suspect Lesions on Diagnostic Accuracy in Melanoma: A Randomized Clinical Trial

**DOI:** 10.1001/jamadermatol.2022.1570

**Published:** 2022-06-01

**Authors:** Giovanni Pellacani, Francesca Farnetani, Silvana Ciardo, Johanna Chester, Shaniko Kaleci, Laura Mazzoni, Sara Bassoli, Alice Casari, Riccardo Pampena, Marica Mirra, Michela Lai, Serena Magi, Victor D. Mandel, Sergio Di Matteo, Giorgio Lorenzo Colombo, Ignazio Stanganelli, Caterina Longo

**Affiliations:** 1 Department of Dermatology, University of Modena and Reggio Emilia, Modena, Italy; 2 Dermatology Clinic, Sapienza University of Rome, Rome, Italy; 3 Department of Surgery, Medicine, Dental Medicine and Morphological Sciences, University of Modena and Reggio Emilia, Modena, Italy; 4 Skin Cancer Unit, Istituto Scientifico Romagnolo per lo Studio dei Tumori (IRST) IRCCS, Meldola, Italy; 5 Centro Oncologico ad Alta Tecnologia Diagnostica, Azienda Unità Sanitaria Locale-IRCCS di Reggio Emilia, Reggio Emilia, Italy; 6 Dermatology Unit, University of Parma, Parma, Italy; 7 Center of Research SAVE Study, Milan, Italy; 8 CEFAT Center of Pharmaceuticals Economics and Medical Technologies Evaluation, University of Pavia, Italy

## Abstract

**Importance:**

Previous systematic reviews and meta-analyses have concluded that given data paucity, a comparison of reflectance confocal microscopy (RCM) with dermoscopy is complex. They recommend comparative prospective studies in a real-world setting of suspect lesions.

**Objective:**

To test the hypothesis that RCM reduces unnecessary lesion excision by more than 30% and identifies all melanoma lesions thicker than 0.5 mm at baseline.

**Design, Setting, and Participants:**

This randomized clinical trial included 3165 patients enrolled from 3 dermatology referral centers in Italy between January 2017 and December 2019, with a mean (SD) follow-up of 9.6 (6.9) months (range, 1.9-37.0 months). The consecutive sample of 3165 suspect lesions determined through dermoscopy were eligible for inclusion (10 patients refused). Diagnostic analysis included 3078 patients (48 lost, 39 refused excision). Data were analyzed between April and September 2021.

**Interventions:**

Patients were randomly assigned 1:1 to standard therapeutic care (clinical and dermoscopy evaluation) with or without adjunctive RCM. Information available guided prospective clinical decision-making (excision or follow-up).

**Main Outcomes and Measures:**

Hypotheses were defined prior to study initiation. All lesions excised (baseline and follow-up) were registered, including histopathological diagnoses/no change at dermoscopy follow-up (with or without adjunctive RCM). Number needed to excise (total number of excised lesions/number of melanomas) and Breslow thickness of delayed diagnosed melanomas were calculated based on real-life, prospective, clinical decision-making.

**Results:**

Among the 3165 participants, 1608 (50.8%) were male, and mean (SD) age was 49.3 (14.9) years. When compared with standard therapeutic care only, adjunctive RCM was associated with a higher positive predictive value (18.9 vs 33.3), lower benign to malignant ratio (3.7:1.0 vs 1.8:1.0), and a number needed to excise reduction of 43.4% (5.3 vs 3.0). All lesions (n = 15) with delayed melanoma diagnoses were thinner than 0.5 mm.

**Conclusions and Relevance:**

This randomized clinical trial shows that adjunctive use of RCM for suspect lesions reduces unnecessary excisions and assures the removal of aggressive melanomas at baseline in a real-life, clinical decision-making application for referral centers with RCM.

**Trial Registration:**

ClinicalTrials.gov Identifier: NCT04789421

## Key Points

**Question:**

Can reflectance confocal microscopy improve diagnostic accuracy for suspect lesions identified with dermoscopy?

**Findings:**

In this randomized clinical trial of 3165 patients, adjunctive use of reflectance confocal microscopy reduced the number of unnecessary excisions by 43.4%.

**Meaning:**

Adjunctive use of reflectance confocal microscopy for suspect lesions improves in vivo diagnoses, reducing the number of lesions excised and effectively identifying invasive melanomas at baseline.

## Teaser

This randomized clinical trial tests the hypothesis that reflectance confocal microscopy reduces unnecessary lesion excision by more than 30% and identifies all melanoma lesions larger than 0.5 mm at baseline.

## Introduction

Diagnostic efforts for melanoma detection focus on early and precise diagnoses, recognized as the greatest prognosis and most economic solution for melanoma.^1^ Dermoscopy is more accurate than the naked eye^2^ but is limited by numerous unnecessary excisions.^3,4^ The rate of benign lesions excised for every melanoma detected range from 5 to 30 lesions, depending on specialization.^5,6^ Specificity is improved with dermoscopy digital follow-up (DDF), with rates of melanoma diagnosis of 7% during monitoring.^7,8^

Reflectance confocal microscopy (RCM) enables in vivo cutaneous examination,^9^ high diagnostic accuracy,^10,11,12^ and specificity improvements for equivocal lesions (30%-70%).^13^ Additionally, RCM appears more accurate than use of dermoscopy alone (specificity, 82% vs 42%)^14^ and improves the accuracy of benign recognition for equivocal lesions.^15^

Skin cancer management exerts a sizable burden on health systems.^16^ The systematic application of RCM in the triage of high-risk patients should improve diagnostic accuracy and reduce unnecessary excisions for histopathological diagnostic confirmation, thereby reducing costs, surgical waiting lists, and delayed diagnoses. However, the clinical application of RCM has mainly been limited to retrospective and prospective observational studies producing hypothetical estimates of clinical applicability without intention to affect clinical and therapeutic patient pathways.^9,11,13,17,18,19,20^

Defined prior to study initiation, this trial hypothesized that adjunctive use of RCM reduces unnecessary excisions by 30% and, among lesions assigned to DDF, melanoma is identified in less than 2% with a Breslow thickness of 0.5 mm or thinner. This study aims to assess adjunctive RCM imaging among randomly assigned equivocal lesions suspected of melanoma to either standard therapeutical care alone or with the integration of RCM, with or without DDF and RCM monitoring. Assessment includes rates of detection, in terms of the number needed to excise (NNE), rates of accuracy, delayed diagnosis, and melanoma Breslow thickness of lesions excised during DDF, based on prospective, clinical decision-making.

## Methods

### Study Design

This prospective, multicenter, 2-arm, randomized interventional study was conducted at 3 Italian centers: the Department of Dermatology of the University of Modena and Reggio Emilia, the Skin Cancer Unit of IRCCS Reggio Emilia, and the Skin Cancer Unit of IRCCS IRST Romagna. The study was approved by the Italian Ministry of Health and the Modena Ethics Committee, and a short, translated version of the study protocol is available in Supplement 1. This study also followed the Consolidated Standards of Reporting Trials (CONSORT) reporting guidelines.

Patients’ access and clinical therapeutical pathways were similar at each collaborating center, per national and regional health care regulations. Briefly, the usual patient pathway includes access to participating dermatological units by referral from other specialists or family physicians. Standard therapeutic care includes clinical and/or dermoscopic assessment by dermatologists. In the case of equivocal lesions, defined as clinically and/or dermoscopically suspected of melanoma owing to the absence of unequivocal clinical and/or dermoscopic aspects of malignancy impeding differential diagnosis of melanoma, patients are invited to adjunctive RCM imaging for prospective decision-making; the patient is accompanied by the visiting dermatologist to a separate, dedicated consultation room with an RCM technician who performs lesion assessment. Images of the skin morphology are assessed on the digital screen in real time by the dermatologist. Based on RCM features observed, the dermatologist decides whether the lesion should be sent for immediate excision or referred to DDF.

For the current study, the patient pathway was modified between August 2017 and June 2019, and consecutive patients with equivocal lesions detected during standard therapeutic care, who provided written informed consent, were then randomly assigned to either access RCM (adjunctive RCM) or not (standard therapeutical care only) at a ratio of 1:1. Unequivocal lesions (benign or malignant), hyperkeratotic or fully ulcerated lesions, and/or lesions located on mucosa or palmoplantar areas or in skin folds not permitting the use of RCM were not included. According to patient randomization, the physician’s management decision (surgical excision or DDF) was based on standard therapeutic care and RCM or standard therapeutic care only. Lesions deferred to DDF were assessed according to standard therapeutic care with or without adjunctive RCM use at the discretion of the physician, independent of initial randomization.

Following randomization, patients who refused excision were included in economic analyses (intention to treat) but could not be included in diagnostic analyses. Patients lost to follow-up were not included in any analyses (Figure).

**Figure.**
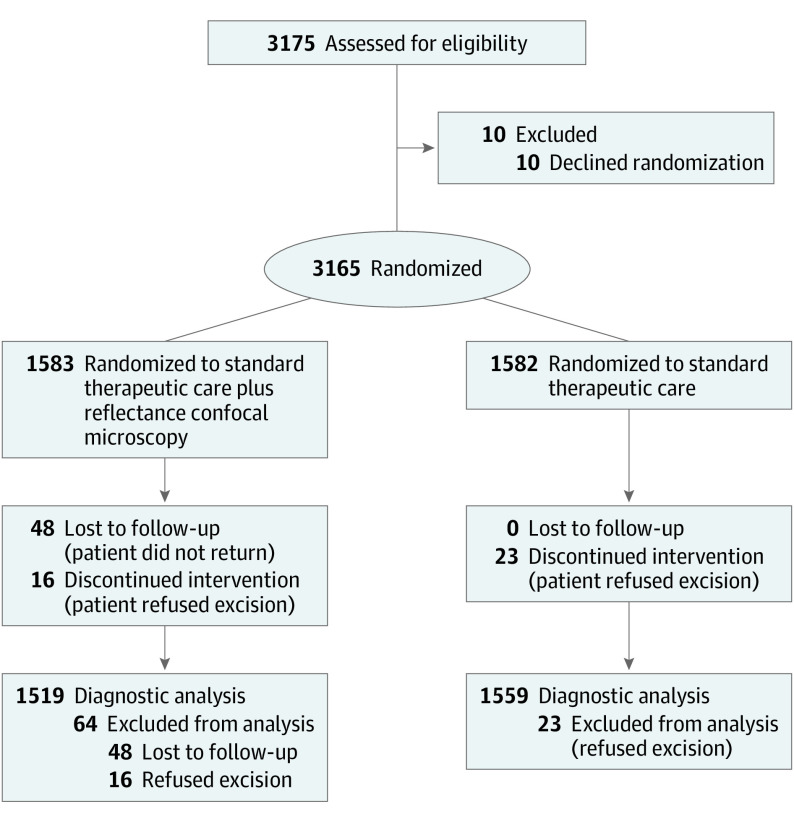
CONSORT Flow Diagram

### Data Collection

All collaborating centers were equipped with a handheld dermatoscope (DL4 [DermLite]), digital dermatoscope (Visiomed [Canfield Scientific]), and reflectance confocal imaging system (VivaScope 1500 [MAVIG GmbH]). Acquisition procedures have been extensively described elsewhere.^21^ Briefly, series of 3 or more mosaic images, including 80% or more of the lesion at depths between 10 and 40 μm below the skin surface (intraepidermal, dermal-epidermal junction, and upper dermis), were obtained. Complete scanning of each lesion took approximately 7 minutes. In case of larger lesions (>1 cm on major axis), a second series of mosaics was recorded to cover the entire lesion. Images were captured by a dedicated RCM technician at each participating center and interpreted in real time on the digital screen by the dermatologist who performed the clinical/dermoscopy consultation. In cases of doubt, colleagues may have been asked for immediate consultation for the development of a collegial decision.

For lesions without unequivocal RCM features determining the need for excision, physicians referred patients to DDF. The scheduled time for DDF reflected the physicians’ level of uncertainty: short (3-6 months) or long (≥12 months). The DDF included standard therapeutic care, and if the physician was still uncertain, RCM evaluation was performed. The DDF images were paired and compared with baseline images; lesions with considerable clinical and/or dermoscopic changes were referred for excision. Lesions without changes at DDF were either rescheduled for subsequent follow-up or considered benign. Final follow-up was completed in March 2021.

Lesion images captured throughout the study period were maintained in both a dedicated database and the centers’ registries of lesions of interest. Therefore, lesions were available for other research analyses.

Histopathologic examination of all excised lesions was performed at the pathology department of the referral center. In case of uncertainty, digitized histopathological samples (D-Sight [Menarini Diagnostics]) were transferred to the pathologists of the other centers and collegially discussed.

The primary outcome was NNE. Secondary outcomes included assessments of diagnostic detection and accuracy at baseline and among lesions assigned to DDF.

### Data Analysis

Data were prospectively maintained in dedicated, clinic-specific databases and united for final analysis at the referral center following study closure. Lesions were grouped as malignant (melanocytic or nonmelanocytic) or benign (melanocytic, nonmelanocytic, or inflammatory). We considered a lesion malignant if histopathological analysis returned a diagnosis of melanoma/melanoma in situ (MIS) (melanocytic) or basal cell carcinoma/squamous cell carcinoma (nonmelanocytic). We considered a lesion benign for diagnoses of nevus (melanocytic), solar lentigo, seborrheic keratosis, lichen planus–like keratosis, lichen simplex (nonmelanocytic), or other (inflammatory).

### Statistical Analysis

#### Sample Size

We hypothesized a 7.3% proportion of melanoma identified in standard therapeutic care,^22,23^ requiring 285 patients per arm to give 80% power (1-sided type I error of 5%). We also hypothesized a 14% proportion of melanoma identified with adjunctive RCM based on approximately 22.4% of suspect lesions in highly experienced centers being treated,^13^ requiring 420 patients per arm to give 80% power (2-sided type I error of 2.5%). Overall recruitment estimates of 3090 patients or more were calculated. No interim analyses were planned.

#### Randomization

A 1:1 randomization at enrollment was applied (computer-generated list of random numbers) by an independent statistician who was not involved in data collection; study experimenters were not involved in list generation or allocation. Randomization sequence was generated using block allocation with variable sizes (2-4) stratified by center using Stata statistical software, version 12.0 (StataCorp), with “ralloc” command.

Following written patient consent, clinicians referred to the randomization list for registration and randomization sequence. Once patient assignment was confirmed, the patient, clinician, and data analysts were aware of study allocation. The study data set was completed by each participant center, and data quality was independently reviewed by a statistician (S.K.).

#### Statistical Methods

In all calculations, melanoma included both MIS and invasive melanoma. Differences in diagnostic frequencies between groups were calculated and compared using the χ^2^ test. The NNE was calculated by dividing the total number of excised lesions by the number of melanomas. Accuracy assessment included positive predictive values (PPVs), benign to malignant and benign melanocytic to melanoma ratios, and correlations with physicians’ RCM experience (malignant lesions/lesions excised). Tests of proportions and equality of proportions for large-sample statistics were used to compare differences in NNE, based on hypothesized population value for no difference in proportions.

Analyses were performed according to the intention-to-treat principle. Subgroup analyses were summarized using descriptive statistics. Continuous variables were expressed as mean (SD) and compared using unpaired *t* test. Categorical variables were expressed as frequencies and compared using Pearson χ^2^ test. Pearson correlation coefficient was used to assess associations between physicians’ years of RCM experience prior to study initiation and correct identification of malignant lesions at baseline with adjunctive RCM use. A *P* < .05 was considered statistically significant, and *r* > 80% was considered a very high correlation. Statistical analysis was performed using Stata statistical software, version 14 (StataCorp).

## Results

### Patient Enrollment and Assignment

Between January 2017 and December 2019, a total of 3165 patients were randomly assigned to either standard therapeutic care with adjunctive RCM imaging or standard therapeutic care only. Ten patients did not give consent to randomization, directly requesting adjunctive RCM evaluation, and were not included in this analysis (Figure). For enrolled patients without immediate lesion excision, the mean (SD) follow-up was 9.6 (6.9) months (range, 1.9-37.0 months).

Patient enrollment was similar according to collaborating centers: IRCCS Reggio Emilia (n = 1140 [35.1%]), University of Modena and Reggio Emilia (n = 1105 [34.0%]), and IRCCS IRST Romagna (n = 1002 [30.9%]). Dermatologists involved in RCM image interpretation and rendering diagnoses at University of Modena and Reggio Emilia (F.F., S.B., A.C.), IRCCS Reggio Emilia (C.L., R.P., M.L.), and IRCCS IRST Romagna (V.M. and I.S.) actively enrolled patients over the entire study period. The RCM experience among the physicians ranged from 4 to 15 years prior to study initiation. Analysis of patient and lesion characteristics according to collaborating centers did not reveal any statistically significant differences.

Among the 3165 total patients, the mean (SD) age was 49.3 (14.9) years, and a personal history of melanoma was registered for 678 (21.4%) patients. Most lesions were identified on the trunk (n = 2167 [68.5%]), and less than half of all lesions had photodamage immediately around the suspected lesion (n = 1423 [44.9%]) (Table 1).

**Table 1.** Clinical Features for Patients With Equivocal Lesions by Randomization Group

Characteristic	No. (%)		
	Total	Standard therapeutic care + RCM	Standard therapeutic care only
No. of patients	3165	1583	1582
Age, mean (SD) [range], y	49.3 (14.9) [18-96]	49.0 (14.9) [18-93]	49.6 (15.1) [18-96]
Gender			
Female	1557 (49.2)	814 (51.4)	743 (47.0)
Male	1608 (50.8)	769 (48.6)	839 (53.0)
Lesion site			
Head/neck	148 (4.6)	63 (3.9)	76 (4.8)
Trunk	2167 (68.5)	1071 (67.7)	1096 (69.3)
Upper limbs	332 (10.5)	177 (11.2)	155 (9.8)
Lower limbs	527 (16.6)	272 (17.2)	255 (16.1)
History of melanoma			
Personal	678 (21.4)	321 (20.3)	357 (22.6)
Familial	448 (14.1)	222 (14.0)	226 (14.3)
Other cutaneous tumor			
Personal	343 (10.8)	158 (9.9)	185 (11.7)
Familial	127 (4.0)	60 (3.8)	67 (4.2)
Phototype^a^			
1	243 (7.7)	112 (7.1)	131 (8.3)
2	1597 (50.5)	820 (51.8)	777 (49.1)
3	1244 (39.3)	618 (39.0)	626 (39.6)
4	78 (2.5)	31 (1.9)	47 (2.9)
Overall No. of nevi			
<50	1436 (45.4)	738 (46.6)	698 (44.1)
50-100	1041 (32.9)	493 (31.1)	548 (34.6)
>100	688 (21.7)	352 (22.2)	336 (21.2)
Overall No. of atypical nevi			
<3	2285 (72.2)	1143 (72.2)	1142 (72.2)
4-6	467 (14.8)	217 (13.7)	250 (15.8)
>6	413 (13.0)	223 (14.1)	190 (12.0)
Photodamage around suspected lesion	1423 (44.9)	688 (43.5)	735 (46.5)

Abbreviation: RCM, reflectance confocal microscopy.

^a^ Data missing for 3 patients.

Characteristics of patients assigned to adjunctive RCM evaluation are summarized in Table 1. Just fewer than half (n = 720 [45.5%]) were sent for immediate excision. According to the time of the DDF visit, among those sent for short-term follow-up, 101 of 564 (17.9%) were assigned excision compared with 21 of 289 (7.2%) sent for long-term follow-up. Melanoma was confirmed in 278 of 836 (33.2%) excised lesions assessed with adjunctive RCM; 144 of the 278 (51.8%) were classified as MIS (Table 2).

**Table 2.** Baseline and Follow-up Lesion Diagnoses and Melanoma Histopathological Features

Characteristic	No. (%)			*P* value
	Total	Standard therapeutic care + RCM	Standard therapeutic care only	
**Baseline lesion diagnoses**	3165	1583	1582	NA
Histology diagnoses	2276 (71.9)	720 (45.5)	1556 (98.4)	NA
Melanoma	557 (17.6)	263 (16.6)	294 (18.6)	<.001
Malignant, nonmelanocytic^a^	52 (1.6)	15 (0.9)	37 (2.3)	
Benign, melanocytic^b^	1548 (48.9)	414 (26.2)	1134 (71.7)	
Benign, nonmelanocytic^c^	56 (1.8)	14 (0.9)	42 (2.7)	
Benign, inflammatory^d^	63 (2.0)	14 (0.9)	49 (3.1)	
Sent to digital follow-up	856 (27.0)	853 (53.9)	3 (0.2)	NA
No diagnosis (patient excision refusal)	33 (1.0)	10 (0.6)	23 (1.5)	NA
**Baseline melanoma features**	557 (100)	263 (47.2)	294 (52.8)	NA
Melanoma Breslow thickness, mean (SD) [range], mm	0.17 (0.23) [0-0.8]	0.19 (0.24) [0-0.8]	0.15 (0.22) [0-0.8]	.02
0.0	314 (56.4)	136 (51.7)	178 (60.5)	.05
0.1-0.5	187 (33.6)	93 (35.4)	94 (32.0)	
>0.5	56 (10.1)	34 (12.9)	22 (7.5)	
**Follow-up lesion diagnoses**	856 (100)	853 (99.6)	3 (0.4)	NA
Histology diagnoses	116 (13.6)	116 (13.6)	0	NA
Melanoma	15 (1.8)	15 (1.8)	0	NA
Malignant, nonmelanocytic^a^	1 (0.1)	1 (0.1)	0	NA
Benign, melanocytic^b^	95 (11.1)	95 (11.1)	0	NA
Benign, nonmelanocytic^c^	3 (0.4)	3 (0.4)	0	NA
Benign, inflammatory^d^	2 (0.2)	2 (0.2)	0	NA
No morphological changes at digital follow-up	686 (80.1)	683 (80.1)	3 (100)	NA
No diagnosis	54 (6.3)	54 (6.3)	0	NA
Patient excision refusal	6 (0.7)	6 (0.7)	0	NA
Lost to follow-up	48 (5.6)	48 (5.6)	0	NA
**Follow-up melanoma features**	15 (100)	15 (100)	0	NA
Melanoma Breslow thickness, mean (SD) [range], mm	0.16 (0.19) [0-0.5]	0.16 (0.19) [0-0.5]	NA	NA
0.0	8 (53.3)	8 (53.3)	0	NA
0.1-0.5	7 (46.7)	7 (46.7)	0	NA
>0.5	0	0	0	NA
Follow-up, mean (SD) [range], mo	9.6 (6.9) [1.9-37.0]	9.6 (6.9) [1.9-37.0]	8 (3.4) [6.0-12.0]	NA

Abbreviations: NA, not applicable; RCM, reflectance confocal microscopy.

^a^ Basal cell carcinoma, squamous cell carcinoma, Bowen disease, and keratoacanthoma.

^b^ Nevus.

^c^ Solar lentigo, seborrheic keratosis, lichen planus–like keratosis, and lichen simplex.

^d^ Others.

Among the 1582 patients assigned to standard therapeutic care only, all lesions with the exception of those in 3 patients who refused surgery were assigned excision (n = 1579 [99.8%]). Of these lesions, 294 (18.6%) were diagnosed through histopathology as melanoma, and then 178 (60.5%) of these were classified as MIS (Table 2).

### Lesions Excised, Excision Ratios, and NNE

Of all 3165 excised lesions, melanoma was identified in 572 (23.9%). The overall study NNE was 4.2. The overall PPV of an excised lesion being melanoma was 23.9% (Table 3). Physicians’ years of RCM experience correlated very highly with diagnostic accuracy (*r* = 0.99; 95% CI, 0.82-0.99; *P* = .004).

**Table 3.** All Lesions Excised at Baseline and Follow-up With Excision Ratios and NNE

Characteristic	No. (%)		
	All lesions	Standard therapeutic care + RCM	Standard therapeutic care only
No. of patients	3165	1583	1582
Lesions excised	2392 (75.6)	836 (52.8)	1556 (98.4)
Melanoma	572 (23.9)	278 (33.2)	294 (18.9)
Malignant, nonmelanocytic	53 (2.2)	16 (1.9)	37 (2.4)
Benign, melanocytic	1643 (68.7)	509 (60.9)	1134 (72.9)
Benign, nonmelanocytic	59 (2.5)	17 (2.0)	42 (2.7)
Benign, inflammatory	65 (2.7)	16 (1.9)	49 (3.1)
Positive predictive value	23.9	33.3	18.9
Ratio			
Benign to malignant	2.8:1.0	1.8:1.0	3.7:1.0
Benign, melanocytic to melanoma	2.9:1.0	1.8:1.0	3.9:1.0
NNE^a^	4.2	3.0	5.3

Abbreviations: NNE, number needed to excise; RCM, reflectance confocal microscopy.

^a^ NNE was reduced by 43.2% with adjunctive use of RCM.

When compared with standard therapeutic care only, the adjunctive use of RCM revealed a slightly inferior rate of melanoma detection (294 vs 278), an almost 2-fold higher PPV (18.9 vs 33.3), and an almost halved benign to malignant ratio (3.7:1.0 vs 1.8:1.0). The NNE was reduced by 43.2% with adjunctive use of RCM (5.3 vs 3.0) (Table 3).

### Diagnostic Safety

Overall, 15 of 853 (1.8%) lesions referred for DDF were revealed as melanoma. Of these, 8 (53.3%) were diagnosed as MIS, and no melanomas identified at DDF were thicker than 0.5 mm. Furthermore, the mean thickness of melanomas identified at follow-up was inferior to baseline values. Over the mean follow-up of 9.6 months, of the 3165 total patients, 39 (1.2%) refused to excise and 48 (1.5%) were lost to follow-up (Table 2).

## Discussion

This randomized interventional trial assessed the applicability of adjunctive RCM for equivocal lesions suspected of melanoma in a clinical setting and proves that unnecessary excisions can be reduced by almost half, with greater accuracy of in vivo identification of benign lesions. Furthermore, delayed diagnosis included thin melanomas only.

Data from this study are essential for the ongoing discussion regarding the applicability of advanced technologies in routine melanoma detection among equivocal lesions. Our group previously assessed the integration of RCM into a diagnostic-therapeutic workflow (with centralized and immediate assessment of suspect lesions) and specific education over a 10-year period in a single province, reporting improved precision in diagnosis of approximately 100%.^20^ Meta-analyses have reported estimates of improved specificity of RCM compared with dermoscopy. In 2020, Pezzini et al^17^ concluded that independent of study design, RCM has a high diagnostic power for melanoma detection (pooled sensitivity of 92%) and reduces unnecessary excisions (pooled specificity of 70%). Recent estimates propose 7.5 benign pigmented lesions are removed for each histologically confirmed melanoma.^24^ Petty et al^6^ studied the NNE for dermoscopy according to clinical setting and found that the NNE was 4-fold lower (5.85) for specialists compared with primary physicians (22.62). In the current study, all diagnoses were performed by specialists and NNE for dermoscopy was 5.3, comparing well with data provided by Petty et al. In a long-term study, Guitera et al^25^ recently reported a rate of 2.4 benign melanocytic lesions biopsied per melanoma for high-risk patients included in a strict DDF. The correlation of detection accuracy with physicians’ RCM experience suggests that, as with dermoscopy, prospective management decision-making is dependent on RCM experience.

Prior to this study, most estimates of NNE calculations were based on retrospective analyses. As suggested by Privalle et al,^26^ retrospective studies based on NNE do not fully assess diagnostic accuracy because they give no insight into the number of malignant lesions that go undiagnosed and they do not consider patient preference for biopsy. This prospective, randomized study reports a slightly higher number of melanomas identified with standard therapeutic care. This minimal imbalance in diagnostic accuracy may be explained by false-positive histopathological results being more likely among higher volumes of excised lesions,^14^ potential thin melanomas undiagnosed among patients lost to follow-up, or false-negative results with RCM assessment. This study also reports prospective rates of deferred melanoma diagnoses, refusal to excise, and loss to follow-up, which are useful for procedure risk assessment.

In this study, RCM assisted in the identification of melanomas with similar mean Breslow thickness compared with standard therapeutical care while requiring just more than half of the number of excisions. Several meta-analyses have proven the advantage of dermoscopy and RCM in the diagnosis of melanoma and nonmelanoma skin neoplasms.^3,27,28,29,30^ However, studies have mostly included heterogenous populations and analyses, potentially providing bias in diagnostic comparisons and rates of unnecessary excisions.^14^ Dinnes et al^14^ concluded that RCM was promising among equivocal lesions, and despite a generally high sensitivity across studies, there was considerable heterogeneity in specificity and studies were generally at high or unclear risk of both bias and concern regarding applicability. Recommendations for future studies included recruitment of prospective and consecutive participants with equivocal lesions ascertained at dermoscopy, assessed and interpreted by RCM in a standard health care setting within multicenter approach, including systematic follow-up of nonexcised lesions.^14^ This prospectively randomized clinical study was designed according to recommendations and proves that, within a homogenous clinical setting, physicians’ diagnostic accuracy is much improved with most melanomas excised at baseline, and those with delayed diagnoses were mainly MIS.

In an era of economic austerity, there is an urgent need for efficient health care services.^16^ Ferris^31^ suggested that cost-benefit analysis of sophisticated technology should ideally be assessed among high-risk patients randomized to receive intensive vs traditional surveillance. Data from the present study will be applied to an independent, separate cost-benefit analysis in response to this request.

### Limitations

This study does not address issues of overdiagnosis associated with early melanoma detection.^32,33^ Furthermore, applicability of this trial is limited to referral centers with RCM experience, but future application of RCM into a general dermatology setting (not specialized clinics) may decrease morbidity among suspect lesions following adequate training. The accuracy analyses related to RCM experience includes a subset of 800 excised lesions with physician name recorded (other data were not recorded). This study does not consider quality of life or reduced surgical waiting lists. Finally, the results of this study cannot be attributed to RCM alone because the patient pathway for those without immediate excision foresaw additional dermoscopy and occasional RCM assessments.

## Conclusions

This randomized clinical trial confirms improved physicians’ diagnostic accuracy with adjunctive RCM. Most melanomas are correctly identified at baseline and very few thin melanomas are identified during digital monitoring.

## Notes

Supplement 1. Trial Protocol

Supplement 2. Data Sharing Statement
