# Supplementary material for: Effect of Reflectance Confocal Microscopy for Suspect Lesions on Diagnostic Accuracy in Melanoma: A Randomized Clinical Trial
Source: JAMA Dermatol. 2022 Jun 1;158(7):754–61. doi: 10.1001/jamadermatol.2022.1570 (PMC9161119; doi:10.1001/jamadermatol.2022.1570)
Supplement: Supplement 1. — Trial Protocol [file jamadermatol-e221570-s001.pdf]

USE OF ADVANCED, NON-INVASIVE DIAGNOSTIC METHODOLOGY FOR EARLY DIAGNOSIS OF SKIN CANCER IN IMPROVING THERAPEUTIC AND ECONOMIC-MANAGEMENT OF CLINICAL PRACTICE.

Background and rationale:

The clinical diagnosis of cutaneous melanoma is complex. The aim of this project is to assess the effectiveness of the systematic integration of reflectance confocal laser microscopy (RCM) into the skin neoplasms diagnostic/clinical pathway.

Two instruments will be assessed:

- fixed head RCM (VivaScope 1500), capable of producing a virtual, non-invasive skin cancer in-vivo biopsy
- manual RCM (VivaScope 3000), for rapid screening of patients at high risk of melanoma for especially for early diagnosis of thin melanomas.

The evaluation of the cost-utility ratio of the device integration will be calculated based on a model specifically designed, developed and tested based on study data, within the network of the 3 collaborating hospitals (in a single Italian Region). The aim is to therefore evaluate whether the proposed skin cancer triage system is useful for reducing waiting lists and costs related to surgery and histopathology.

Skin neoplasms are the most common cancers in humans and represent substantial social costs for diagnosis and treatment. Due to the difficulty in arriving to a diagnosis based on clinical and dermoscopic evaluation only, a ratio of about 30 benign lesions excised for every melanoma identified has been calculated. RCM allows a non-invasive virtual biopsy of the tumor and a more accurate diagnosis thanks to the visualization of the skin lesion at a cytological resolution level. However, the advantage in clinical practice for patients and in economic terms of the implementation of this non-invasive technique as a pre-surgical diagnostic step has not yet been demonstrated. RCM may lead to the early diagnosis of skin tumors otherwise unable to be diagnosed in clinical and dermoscopy evaluations, potentially offsetting the enormous costs of treating advanced disease and considerably reducing the number of unnecessary excisions thus, representing a substantial economic advantage for health care.

RCM is a rapid, inexpensive and single-operator tool which will be added to the diagnostic triage. The main objective of the project is to assess the impact of the systematic application of RCM in a skin cancer triage, in terms of the number of unnecessary excisions, histopathological examinations and overall health costs.

Of interest, particular attention will be paid to the dermatological triage with RCM integration of the head / neck sites. Pigmented spots are often observed in the head / neck area, which are often the font of a high rate of dermatological consultation requests, both for diagnostic evaluation of suspicious lesions and aesthetic treatments. Among these lesions, differential diagnosis between benign and malignant lentigos is challenging. Improper treatment (cryotherapy, laser, radiofrequency, etc.) of an unidentified/misdiagnosed lentigo maligna (melanoma in situ) can induce progression into invasive melanoma, with worsening prognosis. As the evaluation of equivocal lesions is often difficult with dermoscopy alone, a biopsy is usually performed for diagnostic or preoperative planning motives. RCM has been successfully used for in vivo characterization of flat skin facial lesions by enabling the exploration of morphological and architectural lesion aspects suggestive of malignancy. Based on preliminary data available in literature, RCM may potentially positively impact not only healthcare costs, but also surgical planning,

which are especially important for this specific skin topographic area, given the important aesthetic impact of facial surgery.
Finally, the effectiveness of the integration of RCM to the clinical practice in non-melanoma malignancies diagnostics, particularly for basal cell carcinoma will also be assessed. Basal cell carcinoma represents the most common malignant neoplasm in Caucasian and Italian populations (AIRTUM data) and has a significant impact in health management and related National Health System costs. Recently, RCM has been shown to have very high sensitivity and specificity for basal cell carcinoma diagnosis and has therefore been adopted in pre- and intra-operative management of basal cell carcinomas surgical margins verifications, particularly for areas at high risk of local recurrence.

Scope of the Project

Principal Objective

-Evaluate cost-benefit deriving from the presumed reduced number of unnecessary excisions given the accuracy of diagnosis with RCM.
-develop and test a regional triage system for skin cancers based on a radial hospital network model with the systematic integration of RCM into current clinical practice.
- Evaluate diagnostic sensitivity and specificity in screening patients at high risk of skin cancer with the use of the manual RCM tool.

Experimental design 1 aims at a reduction of over 30% of unnecessary excisions, with less than 2% of melanoma delayed
diagnosis (compared with dermoscopy delayed melanoma diagnosis ranging from 7.6 to 16.2%, for digital monitoring (10)),
and with no tumors thicker than 0.5 mm delayed.

Secondary Objectives

- Evaluate diagnostic accuracy of lentigo maligna with different clinical-instrumental algorithms.
- Define which RCM features distinguish lentigo maligna from benign facial macules, such as solar lentigo, freckles, actinic keratoses and seborrheic keratoses, and to develop an easy-to-use diagnostic algorithm.
- Evaluate the accuracy of basal cell carcinoma diagnosis with RCM.
- Analyze and compare the difference of costs of diagnosis with RCM compared to standard histological examination.
- Compare the time required to perform RCM compared to standard histological examination.

Hypothesis to be tested: the advantage of the integration of RCM to the current clinical-diagnostic pathway (clinical-dermoscopic). Outcomes will be measured in terms of the early diagnosis (number of unnecessary excisions), diagnostic safety (number of melanomas diagnosed), and cost-benefit / efficacy calculation based on the drug model. economic developed within the studio.

Design and description of the study

This is a prospective randomized 2-arm study (performed in 3 hospitals). Consecutive patients with at least 1 lesion suspicious of melanoma (following clinical and dermoscopy evaluation) will be enrolled. Patients will be randomized to the standard therapeutical care ("Clinical and dermoscopy evalution only") or standard therapeutical care + RCM integration ("RCM evaluation + clinical and dermoscopy evaluation") following identification of a suspicious lesion during the standard therapeutical care.

The main objective is to evaluate the safety of the integration of RCM (diagnostic identification of melanomas) and cost-effectiveness in terms of the comparison of the number of excisions needed to identify 1 melanoma (including the follow-up period). A cost-utility model will be developed to quantify the impact of the proposed triage system on health care costs and diagnostic effectiveness. This model will be tested on the study population.
Patients will be selected consecutively among patients with suspected skin cancer. Randomization will be allocated at a 1:1 ratio and patients will be allocated to the "RCM evaluation + clinical and dermoscopy evaluation" or "Clinical and dermoscopic evaluation only" following standard therapeutic care; patients allocated to "Clinical and dermoscopic evaluation only" will likely require immediate biopsy for diagnosis, whereas clinicians of patients allocated to the "RCM evaluation + clinical and dermoscopy evaluation" can decide based on RCM evaluation whether to excise the lesion or refer the patient to subsequent digital follow up. Lesions referred to follow-up will be monitored for at least 6 months, and excised if significant changes are observed on dermoscopy.
In detail, the therapeutic-diagnostic pathway for each study arm are as follows:
a) "RCM evaluation + clinical and dermoscopy evaluation": following routine therapeutic care (clinical-dermoscopic examinations) clinicians of patients with suspicious lesions (at least one major or minor clinical and / or dermoscopic criterion for malignancy, as per inclusion / exclusion criteria) will be able to evaluate the lesions with RCM. A clinical decision (removal or digital dermoscopic follow-up) will be made based on the RCM evaluation.
b) "Clinical and dermoscopic evaluation only": following routine therapeutic care (clinical-dermoscopic examinations) clinicians of patients with suspicious lesions (at least one major or minor clinical and / or dermoscopic criterion for malignancy, as per inclusion / exclusion criteria), will make a clinical decision (removal or digital dermoscopic follow-up) without RCM evaluation.
Each participating center will be responsible for its own case history and clinical decisions. Each center will take care of the collection and storage of data. At interim analysis (18 months) collected data and preliminary results will be evaluated collectively and discussed. The investigators at the participating centers have high-level therapeutic-diagnostic pathways in place, as documented by the numerous publications produced by these centers, however, in case of significant discrepancies among the interim clinical performance data, or if requested by individual investigators, some cases may be evaluated independently by other investigators in order to verify diagnostic agreement. These cases may derive either from direct indications from the investigators or randomly selected from the collected cases.
Images will be anonymized and only essential clinical data (sex / age, any significant present or past pathologies) will be provided to the other evaluators.
The definition of the head / neck lesion subset is as follows:
1. Malignant macules with histological diagnosis of melanoma;
2. Benign macules with histological diagnosis of actinic keratoses, solar lentigos, lichen planus-like keratosis, seborrheic keratoses, freckles and junctional / compound / dermal nevi;
3. Benign macules not subjected to biopsy and with clear and defined parameters of clinical, dermoscopic and confocal microscopy benignity;

The main outcome will be the rate of malignant lesions identified within the "RCM evaluation + clinical and dermoscopy evaluation” compared to “Clinical and dermoscopic evaluation only" study arms.
Sensitivity, specificity, diagnostic accuracy, odds ratios (OR) with the 95% confidence interval will be evaluated. Several algorithms will be developed for the diagnosis of lentigo maligna in consecutive head / neck injuries. In particular, the positive and negative predictive values for each individual parameter analyzed will be measured and the combination of parameters capable of increasing diagnostic accuracy and reducing the number of unnecessary excisions, will be identified.
RCM features will be systematically analyzed to distinguish lentigo maligna from benign facial lentigos.
The time required for the assessment of face / neck injuries will be calculated and compared within the two groups.
The subgroup of non-melanocytic malignant lesions should include consecutive cases defined as:

1. Carcinomatous neoformations with histological diagnosis of basal cell carcinoma
2. Carcinomatous neoformations with histological diagnosis of squamous cell carcinoma
3. Benign neoformations with histological diagnosis of actinic keratoses, solar lentigos, lichen planus-like keratosis, seborrheic keratoses, ephelides and junctional / compound / dermal nevi.
4. Benign neoformations not subjected to histological analysis and with clear and defined parameters of clinical, dermoscopic and RCM benignity.

The main result is to evaluate the rate of histologically confirmed, excised basal cell carcinomas detected in the "RCM evaluation + clinical and dermoscopy evaluation” compared to “Clinical and dermoscopic evaluation only" study arms.
Sensitivity, specificity, diagnostic accuracy, odds ratios (OR) with the 95% confidence interval will be evaluated. Finally, the differences between the time, technical and human resources required within the "RCM evaluation + clinical and dermoscopy evaluation” compared to “Clinical and dermoscopic evaluation only" study arms.

Statistical analysis

Randomization: the “sample” function of STATA 13 (STATA Corp. College Station, Texas 2014) will be used weekly to predetermine the allocation of the subjects, in an alternating random manner, into the two study arms.
In the Coordinating Center, an estimated 800 patients will be enrolled (a total of 400 cases per study arm).
Outcomes include diagnostic safety of the RCM tool (measured by the number of excisions in relation to the number of melanomas diagnosed), a cost-benefit / efficacy calculation (based on an economic-drug model developed within the studio).
The estimated number of delayed cancer diagnosis within the “RCM evaluation + clinical and dermoscopy evaluation” study group is less than 1%.
Study power calculation: The expected percentage of melanomas identified in the "Clinical and dermoscopic evaluation only " group is 7.3% (*Carli P, De Giorgi V, Nardini P, Seidenari S, Pellacani G, Peris K, Piccolo D, Rubegni P, Perotti R. Skin Cancer Day in Italy: tumor detection rate and pattern of referral of open access clinics in an intermediate melanoma risk population. Eur J Dermatol 2003; 13: 76-79*) and for the " RCM evaluation + clinical and dermoscopy evaluation " group is 14% (*Guitera P, Pellacani G, Longo C, Seidenari S,*

Avramidis M, Menzies S. In vivo reflectance confocal microscopy enhances secondary evaluation of melanocytic lesions. J Invest Dermatol 2009; 129: 131-138). To obtain a power of 80% with level $\alpha = 0.05$, the calculated number of subjects is 532.
As outlined in literature, approximately 22.4% of patients with "atypical" lesions are sent for treatment in highly experienced centers, equipped with digital dermoscopy (*Alarcon I, Carrera C, Palou J, Alos L, Malvehy J, Puig S. Impact of in vivo reflectance confocal microscopy on the number needed to treat melanoma in doubtful lesions. Br J Dermatol. 2014; 170: 802-8*) therefore, the enrolment of at least 2375 patients is required. Each collaborating center will enroll about 800 subjects.
The differences in the frequency of diagnoses obtained in the two study groups will be calculated and compared using the X2 test (chi-square). As a measure of diagnostic efficiency, the NNE (Number Needed to Excise), corresponding to the ratio between malignant lesions and excised benign lesions, obtained in the two different arms, will be calculated. Kolmogorov-Smirnov two-sample Z-test will be used to compare the differences in NNE obtained in the 2 arms. The cost - utility model will be developed in collaboration with the University of Pavia, Department of Pharmacological Sciences, in order to quantify the economic impact on the National Health System.
The model will take into account the costs of treatment (surgery / drug), examination, follow-up visits, devices and histopathology evaluation. The model will be constructed considering two possible alternatives: surgical excision versus RCM examination of equivocal lesions. The model will be tested and adapted based on the real data obtained from the prospective study.

Inclusion criteria:

1) Adult patients (18-90 years) referred to the Dermatology departments of the collaborating centers.
2) Patients able to give consent to participate
3) Patients with at least 1 lesion with at least one major or minor clinical and / or dermoscopic criterion of suspicion of malignant tumor lesion (*Friedman 1987; Argenziano 2002*) identified during the clinical and dermoscopy evaluation.
4) Willingness to participate in the study and able to give written informed consent

Exclusion criteria:

1) Patients <18 years old
2) Comorbid dermatological pathologies that can alter the visualization of the lesions in RCM, such as extensive hyperkeratotic skin diseases or widespread eczema.
3) Physiological, general pathological or psychiatric conditions that prevent the patient from being able to lie down for at least 10 minutes (time of RCM evaluation)
4) lesions with alterations (ulceration, crust, hyperkeratosis) which prevent RCM visualization.

Additionally, in a smaller population of patients classified at high risk of melanoma, will be evaluated with a total body examination with the RCM (VivaScope 3000) for each melanocytic skin lesion in addition to classic videomicroscopy. Suspected lesions in dermoscopy and / or RCM will be excised. Subsequently, the accuracy of excisions will be compared for both groups, and the benefit of the combined approach to the patient will be calculated. A population of about 300 patients at high risk of developing melanoma (> 100 nevi,> 4 atypical nevi) will be required. These tests will be performed in two of the collaborating outpatient clinics, performed by 2 interchangeable expert specialists (both will examine 50% of the population with dermoscopy and 50% with RCM). Coincident and

discordant data in case of equivocal injury will be compared. Any lesion showing features in demoscopy and / or confocal that are suspicious of melanoma will be excised. In high-risk patients, any lesions that do not show clear benign features will be routinely monitored with digital videodermoscopy for at least six-months.
The main objective in this case is the quantification of detection of suspected and confirmed malignant lesions on histological examination after excision, with respect to the standard of care (clinical examination + videomicroscopy). The time required for the total-body examination will be measured and compared with a standard videomicroscopy to estimate the cost-benefit value of the procedure.
The previously described statistical tests (chi-square and Z-test) will be applied.

RCM, a non-invasive method performed in vivo, allows an almost histological resolution of skin lesions (*Longo Dermatologia Clin 2012*) used in the standard clinical routine. This method has been used for years in clinical practice at all the collaborating centers. The specific characteristics of melanoma and non-melanoma skin cancer that can be observed with RCM have already been extensively described in literature (*Pellacani JAAD 2005; Pellacani JAAD 2009; Longo Dermatology 2013; Ulrich BJD 2007*).
An increase in diagnostic specificity in skin cancers has already been demonstrated in retrospective studies (*Pellacani JID 2007; Guitera JID 2012*).

Data collection
Patients will be coded on the basis of a code generated for each patient enrolled which includes 2 letters (indicating city of enrollment) and 4 consecutive digits (in order of enrolment). In case of multiple lesions for a single patient, lesions will be assigned a letter (A, B, C etc). Essential clinical information for each patient will be collected on an EXCEL file (Gender, age, phototype, number of nevi, number of atypical nevi, family and / or personal history of melanoma, clinical signs of photodamage). For each lesion, the outcome decided during the visit (excision, follow-up) will be indicated. In the event of a lesion referred for follow-up, the subsequent Clinicians' decision (no further follow-up, further follow-up or excision) will be noted.
In the case of excision, upon histological referral, a coded diagnosis will be entered for all the major diagnostic categories. A different EXCEL file, stored on a separate hard-disk and not connected to an external internet network, will collect the code assigned to the patient, the patient's personal data, the date of the visit, any date on which the follow-up is planned / scheduled and personal contact information (telephone / email, etc.) useful for contacting the patient in case the patient does not present for the scheduled follow-up visit. The images coded as described above, without being able to recognize the patient, will be collected on a local Server, with exclusive access allowed by the investigators, through a personal password.
Image management: the images obtained will be coded on the basis of the code generated for each patient enrolled. The images will be stored on local systems (at each individual collaborating center).
Missing data management: any eventual loss of participation by patients' refusal to participate in the study and generated by the randomization process will be compensated the following week, to assure that the number of the patients within both study groups meet the planned dimensions. In case of patients' refusal for surgical intervention, the subject will remain in the study and be monitored over time with a digital follow-up (routine

clinical practice in the case of patients who refuse intervention for lesions without significant suspicion of malignancy), in order to identify lesion modifications, such as increased suspicion of malignancy, enabling a strong recommendation for lesion excision.

Data ownership

Each investigator will be the owner of the data collected at the individual centers. The experimental data, shared for the realization of the current study, will be the equal property of the three principal investigators.

Each study derived from the data collected, will be shared between the 3 principal and co-investigators prior to being sent to scientific journals for publication.

Expected results, possible problems and solutions

The project aims to prove the reduction of more than 30% of unnecessary excisions, with <2% of melanomas with delayed diagnosis (compared with the delayed melanoma diagnosis associated with dermoscopy, which has an estimated range of 7.6- 16.2%. [*Salerni G et al JEADV 2012*), and no thin tumor (thickness> 0.5mm) diagnosed late. The integration of the RCM tool into the standard care pathway is estimated to demonstrate a possible saving of ~€ 30,000 /100,000 inhabitants / year. The risk of misdiagnosing a true melanoma with RCM is minimized, as patients with clinical/dermoscopically suspicious lesions are referred to digital dermoscopy follow-up, where any changes should be recognized within a short interval of time, without risk to the patient's health. The risk of patient loss to follow-up will be minimized, by the allocation of a follow-up appointment during the initial consultancy visit, with the activation of a dedicated telephone number for enrolled patients who may need to modify their pre-programmed follow-up. Additionally, patients who do not present for the pre-programmed consultancy visit will be contacted by telephone.

The cost-utility model, adaptable to different local situations, will be published and made available for the implementation of similar triage systems according to a radial model for the management of skin neoplasms.

The integration of RCM in the triage of high-risk patients will demonstrate the real advantages, by calculating RCM diagnosed melanomas which may have been misdiagnosed with video microscopy alone, due to the lack of significant dermoscopic features.

The risk of increasing the number of unnecessary excisions will be considered and verified periodically through an interim analysis every 6 months.

Meaning and innovation

If RCM is proven to be an effective tool in avoiding unnecessary excisions, the integration of RCM within a radial model (such as that developed in this study) may be appropriate for the National Health System. The benefits of a more accurate examination of patients at high-risk of melanoma could save the high costs of treating advanced cancers.

The overall impact of this study will be to influence health management for skin cancer patients, by creating a more efficient and less expensive diagnostic triage model. For the Principal Collaborating Center, the research group will be composed of trained personnel (with years of RCM experience and internationally recognized RCM expertise, along with numerous scientific publications in the field of non-invasive oncological diagnostics with dermoscopy and RCM). The Principal Collaborating Center will also coordinate the prospective study on diagnostic efficacy and the cost-benefit estimate for the implementation of RCM in the triage of skin cancers. The collaboration with the Department of Pharmacological Sciences of the University of Pavia will ensure the development of an adequate and dedicated cost-benefit model. The sub-study assessment (with manual RCM) in the early diagnosis of superficial melanomas, will also include a team with a high level of

expertise for both dermoscopy and RCM. The other Collaborating Centers, in addition to participating in the prospective randomized study, will focus on the application of manual RCM in other complex clinical situations, such as the early diagnosis of lentigo maligna and pre-surgical definition of initial basal cell carcinoma. Overall, a complete picture of the advantages and limitations deriving from the use of the manual RCM for cutaneous neoplasm diagnosis will be calculated and an estimate of its cost-utility will be performed.
Resources available for the project

• 3GDermLite manual dermatoscope (2 devices). DermLite is a patented technology dermatoscope that combines non-polarized light dermoscopy for use with refractive liquid and polarized light dermoscopy for use with or without skin contact.
This versatile device offers the ability to screen many lesions in the same patient within seconds.

• Vidix digital videodermatoscope (two devices): this is a digital videodermatoscope that allows the visualization and storage of clinical and dermoscopic images. The camera has all the controls: display, photograph images, select magnifications, modify the acquisition parameters, modify the brightness, allow the passage from dermoscopy mode to the clinic and vice versa. This digital dermatoscope has several advantages: a digital camera with autofocus and automatic zoom and the ability to acquire images with different magnifications from 7x to 100x (clinical image, polarized image and magnifications), has a new, more intuitive software for faster and more accurate management of patient images and archives.

• VivaScope 1500 ® RCM (1 device): The VivaScope 1500 is an industry-leading in vivo RCM device for the observation and capture of in vivo images. The instrument is paired with a VivaCam ® dermoscopic digital camera. When recording with VivaScope 1500, the captured dermoscopic images can be used to precisely navigate the area of interest. The camera integrated within the VivaScope 1500 allows you to capture and record clinical photographs of an area of interest, onto which a map of the areas analyzed can be nominated.

Conclusion

This study aims to define an ideal triage for skin cancer patients within a Regional radial system, with reference centers equipped with RCM, able to perform virtual biopsies in high-risk patients before and/or instead of surgical excision.

The increased diagnostic accuracy should be useful in reducing waiting lists and offering cost savings related to reduced surgery / histopathology procedures. The data will produce guidelines based on the evidence-based medicine model, the cost-benefit model and the risk analysis for the integration of RCM in the clinical diagnosis phase of skin cancer.
