# Supplementary material for: Effect of Reflectance Confocal Microscopy for Suspect Lesions on Diagnostic Accuracy in Melanoma: A Randomized Clinical Trial
Source: JAMA Dermatol. 2022 Jun 1;158(7):754–61. doi: 10.1001/jamadermatol.2022.1570 (PMC9161119; doi:10.1001/jamadermatol.2022.1570)
Supplement: Supplement 2. — Data Sharing Statement [file jamadermatol-e221570-s00.pdf]

Data Sharing Statement

Pellacani. Effect of Reflectance Confocal Microscopy for Suspect Lesions on Diagnostic Accuracy in Melanoma. *JAMA Dermatol*. Published June 01, 2022.
doi:10.1001/jamadermatol.2022.1570

Data
Data available: Yes
Data types: Participant data with identifiers
How to access data: Data will be made available for any reasonable request made to pellacani.giovanni@gmail.com
When available: With publication

Supporting Documents
Document types: None

Additional Information
Who can access the data: Data will be made available to researchers whose proposed use of the data has been approved
Types of analyses: for any purpose
Mechanisms of data availability: following approval of a proposal
